# Concurrent Down-Regulation of PTEN and NKX3.1 Expression in Iranian Patients with Prostate Cancer

**DOI:** 10.1590/S1677-5538.IBJU.2014.0036

**Published:** 2015

**Authors:** Vahideh Nodouzi, Mohammadreza Nowroozi, Mehrdad Hashemi, Gholareza Javadi, Reza Mahdian

**Affiliations:** 1 Department of Biology, Science and Research Branch, Islamic Azad University, Tehran, Iran; 2 Urooncology Research Center, Tehran University of Medical Sciences, Tehran, Iran; 3 Department of Genetics Tehran Medical Branch, Islamic Azad University, Tehran, Iran; 4 Department of Molecular Medicine, Pasteur Institute of Iran, Biotechnology Research Center, Tehran, Iran

**Keywords:** Prostatic Neoplasms, NKX3-1 protein, human [Supplementary Concept], PTEN Phosphohydrolase, Real-Time Polymerase Chain Reaction, Biological Markers

## Abstract

NKX3.1 and PTEN genes are involved in the development and progression of prostate cancer (PCa). Here, in line with other studies that correlated the expression of these two genes, we aimed at evaluating the expression pattern of these genes in clinical PCa samples. Collectively, 81 tissue samples including 45 human PCa and 36 benign prostatic hyperplasia (BPH) specimens were included in the study. The tissue samples were subjected to RNA extraction and subsequently to cDNA synthesis according to the kit manufacturer's protocol. Quantitative Real-Time PCR assay was performed for each sample in triplicate reactions. REST and SPSS software were used to statistically analyze PTEN and NKX3.1 gene expression data.

Expression level of both NKX3.1 and PTEN genes was down-regulated in PCa samples compared to BPH samples. The relative expression ratio of PTEN and NKX3.1 was decreased to 0.155 and 0.003, respectively (P=0.000). The results of Chi-Square analysis revealed a significant correlation between the expression of these genes in both BPH and cancer groups (P=0.004 and 0.001, respectively).

According to previous studies and our data, we concluded that the association between the down-regulation of PTEN and NKX3.1 genes contributed to the prostate tumorigenesis. This might highlight the interaction between the proteins encoded by these genes. Furthermore, this finding might be exploited for the development of innovative diagnostic and therapeutic approaches in PCa.

## INTRODUCTION

Prostate cancer (PCa) is the most commonly diagnosed cancer worldwide. It is the second most common type of cancer in men and causes the death of approximately 250,000 men annually (1). PCa is a heterogeneous disease with variable clinical behavior. This heterogeneity increases significantly with progression from benign to malignant form (2). Since there are no effective therapeutic options for advanced PCa, the identification of high risk individuals and early detection of the tumor when it is still confined to the prostate tissue are highly desired.

Although different grades of PCa (including prostatic intraepithelial neoplasia, invasive adenocarcinoma and metastatic forms) have been well defined histologically (3), molecular mechanisms involved in the progression of the disease have not been fully described yet. Recently, developments in molecular genetics techniques have led to the identification of more than 200 genes related to PCa. These genes are predominantly expressed in PCa epithelial cells and affect the initiation and progression of PCa.

NKX3.1 is an androgen-regulated homeodomain gene, whose expression is restricted to the prostate epithelium (4). As a prostate-specific transcription factor with relative molecular mass of 26 kDa, NKX3.1 is necessary for normal development and function of the prostate (5). NKX3.1 gene is affected by the loss of heterozygosity in 60–80% of prostate carcinomas (6), whereas no point mutations were observed in its coding sequence (7). The loss of a single allele of the gene may be sufficient to promote prostate carcinogenesis in humans, confirming haploinsufficiency for this phenotype (8). So far, several mechanisms have been proposed for the loss of NKX3.1 expression in human PCa, including both transcriptional and post transcriptional modifications as well as epigenetic regulation and protein degradation (9).

PTEN was first identified as a tumor suppressor gene in 1997 (10, 11). PTEN gene is located on chromosome 10q23 and encodes an amino acid sequence with relative molecular mass of 47 kDa (10). It is mostly expressed in brain, colon, breast as well as gastric and prostate epithelial cells. After P53, PTEN is the second most mutated tumor suppressor gene. It is frequently inactivated as a result of loss of heterozygosity in up to 70% of primary PCa cases (11, 12).

PTEN contributes as a hub protein in cellular pathways, such as angiogenesis, apoptosis, cell cycle and cell migration. Moreover, it is frequently inactivated in somatic cancers such as PCa (12). Homology of tyrosine phosphatase domain of PTEN to tensin protein suggests that PTEN may suppress tumor cell growth. This activity is accomplished by antagonizing protein tyrosine kinases. Hypothetically, PTEN can regulate tumor cell invasion and metastasis by arrested angiogenesis, which is required for cancer growth and metastasis. This effect is mediated by blocking the transcription of VEGF gene (13). All these effects are likely mediated via PIP3 hydrolysis by PTEN (14).

Some studies have indicated that loss of PTEN function correlates with the decreased expression of NKX3.1 and PCa progression in both mice and humans (15–17). A pioneering study showed that PTEN controls the activity of NKX3.1 through the regulation of its expression (16). The exogenous up-regulation of NKX3.1 obviously blocked the proliferative and anti-apoptotic effects of PTEN loss in PCa cells. Furthermore, the mice compound heterozygous for NKX3.1 and PTEN gene deletion showed fast progression to invasive and androgen independent disease (17).

In this study, we aimed to evaluate the changes in the pattern of NKX3.1 and PTEN gene expression and their contribution in the prostate tumorigenesis in Iranian PCa patients.

## MATERIALS AND METHODS

### Sample collection

Prostate tissue samples, including both tumor and benign prostatic hyperplasia (BPH) samples were selected from patients who were referred to the urology department at Uro-oncology Research Center (UORC) at Clinical Research Development Center (Tehran, Iran) from June 2011 to February 2013. A written informed consent was obtained from each participating patient. The study was also approved by the Ethics and Clinical Research Committee of Pasteur Institute of Iran (Tehran, Iran). Relevant clinical and pathological data were collected for the patients. All patients were new cases with no medical history of surgery or chemotherapy. The median age of the patients was 63 years, ranging from 47 to 75. The patients were examined by an expert urologist and evaluated according to standard imaging procedures and laboratory analyses for PCa. The studied samples consisted of 81 tissue samples including 45 PCa and 36 BPH specimens. The median age of the patients was 68 years, ranging from 49 to 83 years. The mean plasma PSA level was 17.82±2.13ng/mL and 7.71±1. 80ng/mL (Mean±SEM) in cancer and BPH groups, respectively. The Gleason score for the PCa tissues varied from 4 to 10. Tissue samples were obtained via open radical prostatectomy or needle biopsy. Each tissue sample was obtained in two replicates; one replicate was examined by a pathologist for the detection of malignant changes and the evaluation of tumor grade as Gleason score. The other replicate was instantly immersed in RNAlater solution (Qiagen, Germany) and kept at room temperature for 24 hours and subsequently stored at −80ºC. Then, the tissue samples were transferred into liquid nitrogen containers for long term storage. Pathological examinations confirmed either the presence of cancerous cells or BPH. Only tissue sections containing at least 80% tumor cells were included in the study.

### RNA extraction and cDNA synthesis

Total RNA was extracted from 80–100mg of tissue samples using TRI reagent (RiboPure™ Kit, Ambion, USA) according to the manufacturer's instructions. The concentration and quality of the purified RNA were determined by spectrophotometry (IMPLEN, Germany). High quality RNA samples (A260/280>1.8) were used as templates for cDNA synthesis. Selected RNA samples were kept at −80ºC for subsequent cDNA synthesis. RNA samples (up to 1μg) were converted to cDNA using Quantitect® Reverse Transcription Kit (Qiagen, Germany) according to the kit instructions. The cDNA integrity was verified by validation experiments using GAPDH specific primers. The cDNA samples with satisfactory quality were diluted in a ratio of 1:10 and stored at −20ºC until use for further analysis.

### Primer design

NKX3.1 and PTEN genes were considered as target genes and PSA (prostate-specific antigen) and GAPDH as reference genes. Primers design was performed by Primer Express V.3.0 (Applied Biosystems, USA) software and verified by Gene runner V.3.05 and Allele ID V. 6 (Biosoft Int.) software. Moreover, BLAST analysis was used to test the specificity of the primers for their targets (http://www.ncbi.nlm.nih.gov/blast). To further improve the accuracy of the quantitation of mRNA expression, the target fragments spanning distinct exon-exon boundaries of the mRNA transcripts were chosen. Primer sequences were synthesized at Bioneer Corporation (South Korea). The sequence of the primers are shown in Table-1.

**Table 1:** Forward and reverse primers for NKX3.1, PTEN, GAPDH and PSA genes.

Gene	Forward primer	Reverse primer	Amplicon (bp)
NKX3.1 3–1 (NM_006167.3)	CCAGAGCCAGAGCCAGAGG	TCCAACAGATAAGACCCCAAGTG	143
PTEN (NM_000314.4)	CACACGACGGGAAGACAAGTTC	CCTCTGGTCCTGGTATGAAGAATG	161
GAPDH NM_006167.3	ACACCCACTCCTCCACCTTTG	TCCACCACCCTGTTGCTGTAG	112
PSA (NM_001648.2)	TCTGCGGCGGTGTTCTGG	GCTGTGGCTGACCTGAAATACC	140

### Quantitative Real-Time PCR

SYBR Green Master Mix was used to perform a 40-cycle PCR reaction using SDS v.1.0.1 software (ABI System 7300, Applied Biosystems, USA) in MicroAmp Optical 96-well plates. Each PCR amplification reaction (20μL) contained 10μL Power SYBR Green PCR Master Mix (2x), 1μL from each forward and reverse primers, 3μL of first-strand cDNA and 5μL D.D.W. Also, non-template control (NTC) reactions were included in all experiments. The thermal-cycling conditions were as follows: stage 1, 1 cycle for 10 min at 95ºC as first denaturation and Hot-start enzyme activation, followed by stage 2, 40 cycles at 95ºC for 15 s and at 60ºC for 1 min as annealing and extension stages. This stage or process was followed by a dissociation stage (95ºC for 15s, 60ºC for 30 s and 95ºC for 15s) to verify the specificity of the PCR products. Analysis of the results confirmed the specific amplification of the interest gene fragments. Each amplicon was identified by its specific melting curve Tm. Additionally, electrophoresis of the PCR products on 2% agarose gel (V: 80, time 45 min) revealed a single sharp band with expected length of 143, 161, 112 and 140 related to NKX3.1, PTEN, GAPDH and PSA, respectively.

### Quantitative data analysis of Real-time PCR

Serial dilutions were prepared as ten-fold dilutions of the target nucleic acid to draw a standard curve by plotting related Ct values against log cDNA concentrations. To analyze the amount of change in gene expression, we used comparative threshold cycle (Ct) and to calculate the ΔΔCt values, mean threshold cycle (mCt) was obtained from triplicate series of amplifications during the exponential phase. Then, mCt value of reference gene (PSA or GAPDH) was subtracted from mCt value of the target gene (NKX3.1 or PTEN) to obtain ΔCt. Subsequently, ΔΔCt values were calculated from corresponding ΔCt values separately for each sample, using the following formula: ΔΔCt=[mCt target-mCt reference] (cancer) - [mCt target - mCt reference] (BPH). Finally, using the ratio formula (ratio = 2-^ΔΔCt^), up or Down-Regulation of target gene was achieved in proportionate to the reference gene.

## RESULTS

### Melting curve analysis and gel electrophoresis

**Figure 1:**
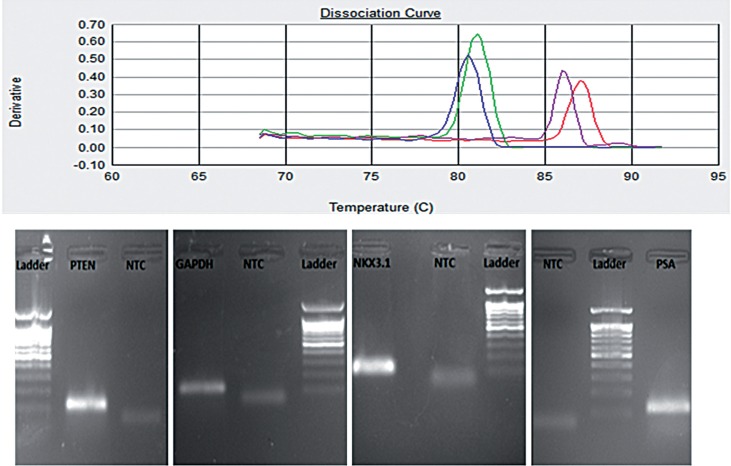
Dissociation curves of PTEN, GAPDH, NKX3.1 and PSA genes drawn through Real-time PCR assay experiments. The corresponding PCR bands revealed by agarose gel electrophoresis are also shown (from left to right).

Based on the temperature and dF/dT derivation, the melting curve was drawn. This curve was made to differentiate between the primer dimmers or non-specific products with the desired amplicons. The melting peaks for PTEN, GAPDH, NKX3.1 and PSA genes have been drawn at 81, 81.5, 85.5 and 87ºC, respectively. Moreover, gel electrophoresis of PCR products showed the specific amplification of the genes of interest (Figure-1).

### Data analysis

**Figure 2:**
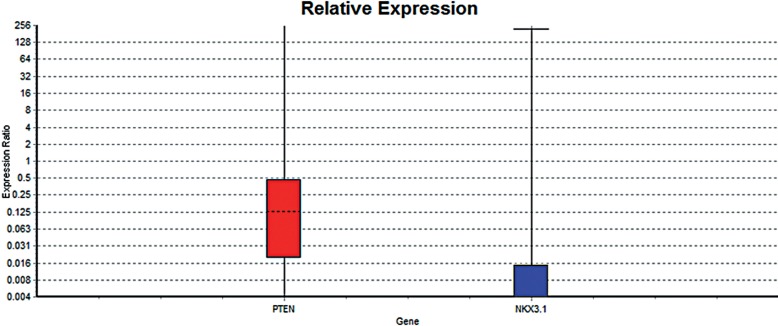
The results of the gene expression analysis obtained by REST software. Box-plots represent the interquartile range or the middle 50% of observations.

Comparison between mean gene expression levels in the study groups (PCa Vs BPH) and graph preparation were performed using Relative Expression Software Tool software (REST© 2009, Qiagen, Germany) (Figure-2). REST© software compares two or more treatment groups or conditions with data points (CT) in sample or control group for multiple reference and target genes. As expected from previous studies (15, 16), the expression level of NKX3.1 gene was significantly different between cancer and BPH group (P=0.000). In fact, the relative expression of NKX3.1 gene was drastically decreased in the PCa patient samples compared to BPH samples (relative ratio=0.003, P=0.000). In addition, similar result was achieved for PTEN gene (relative ratio =0.155, P=0.000) as its relative expression was declined in comparison with BPH samples (Table-2). Interestingly, our data suggested the complete loss of NKX3.1 expression in high-grade and metastatic PCa samples. Also, the consistent alteration in the expression of NKX3.1 and PTEN genes was associated with prostate tumorigenesis. Chi-square analysis (SPSS software, V 16.0) was performed to evaluate the correlation between the expression level of these two genes in the study group. P values<0.05 were considered statistically significant. It showed a significant relation in both BPH (P=0.001) and cancer groups (P=0.004).

**Table 2:** The results of the gene expression analysis by REST software. PTEN is down-regulated in sample group (in comparison to control group) by a mean factor of 0.155 (Std. Error range is 0.033–0.745). PTEN in sample group is different to control group. P (H1) = 0.000. NKX3.1 is down-regulated in sample group (in comparison to control group) by a mean factor of 0.003 (Std. Error range is 0.000–0.026). NKX3.1 sample group is different from control group. P (H1) = 0.000.

	Type	Reaction efficiency	Expression	Std. Error	95% C.I.	P (H1)	Result
PSA, GAPDH	REF	1.0	1.000				
PTEN	TRG	1.0	0.155	0.033–0.745	0.033–0.745	0.000	DOWN
NKX3.1	TRG	1.0	0.003	0.000–0.026	0.000–0.296	0.000	DOWN

## DISCUSSION

There is a general consensus that PCa is the second most common cancer worldwide, causing over 250,000 death in men each year (1). Owing to the fact that there are no effective therapeutic options for advanced PCa, early detection of cancer patients and identification of high risk cases is highly desired. Currently, both PCa diagnosis and management are mainly dependent on serum levels of PSA. In spite of the fact that PSA testing has been used for a long time as a diagnostic aid for PCa detection, it has certain limitations (18, 19). The lack of specificity has been one of these major hurdles resulting in high rate of negative biopsies. Therefore, further investigation is needed to establish more specific and sensitive markers of PCa (18, 20). The identification of PCa specific genes can effectively pave the way for improving the accuracy of PCa diagnostic tests. Achievement of this goal requires the study of gene expression patterns in different stages of PCa from BPH to metastatic PCa (21). More recently, breakthroughs in molecular genetics techniques have contributed to the identification of genes, which are expressed in PCa epithelial cells and related to PCa growth (22).

Alteration in the expression of PTEN has been strongly implicated in PCa development, since mutations in its gene are found in a large proportion of both primary and metastatic PCa (30% and 63%, respectively) (19). Moreover, PTEN mutations are among the most frequent genetic alterations in human PCa (23). Our findings provide new insights into the PTEN role as a biomarker in prostate tumorigenesis. This gene can be included in gene signatures for early diagnosis of PCa in combination to other biomarkers (20).

NKX3.1 is expressed exclusively in prostatic cells and considered as a differentiation-related gene (4). Murine NKX3.1 is the earliest known marker of prostate epithelium during embryogenesis, that subsequently is expressed in all stages of prostate differentiation (24, 25). NKX3.1 protects differentiated prostate epithelium from oxidative DNA damage and inhibits the AKT phosphorylation/activation via an androgen receptor-dependent mechanism (26, 27). Previous studies have shown that NKX3.1 expression is down-regulated during early stages of prostate tumorigenesis (19, 21, 28). Because of its chromosomal localization to a PCa hot spot (minimal region of 8p21), several studies have proposed that NKX3.1 is a prostate specific tumor suppressor gene in which loss of a single allele may predispose to prostate carcinogenesis (4, 6, 8). Interestingly, complete loss of NKX3.1 expression in high-grade tumor samples indicates that it could precisely predict PCa. Despite strong correlation between loss of NKX3.1 expression and PCa initiation and progression, the involved mechanisms are still remained to be described. So far, several mechanisms which contribute to the loss of NKX3.1 in PCa have been proposed, including allelic loss, post-transcriptional control and epigenetic mechanisms (9, 19). However, Lind et al. study on the methylation status of NKX3.1 promoter showed that the promoter was unmethylated (29). In 2010, Jong et al. suggested that NKX3.1 down-regulation is not caused by promoter hypermethylation. They also proposed that other epigenetic mechanisms such as structure modulation of chromatin or histone modifications might be involved (29). In another study, Kunderfranco et al. (30) examined the expression of transcription factor ERG (ETS related gene), NKX3.1 and androgen receptor using immune histochemistry. Surprisingly, they observed that NKX3.1 is directly controlled by ERG in prostate tumors. In a study conducted by Lei et al., it has been suggested that PTEN largely modulates the NKX3.1 function through regulating of its expression (16). They demonstrated that PTEN loss causes reduced NKX3.1 expression in both murine and human PCa. Interestingly, the NKX3.1 restoration can alleviate the adverse phenotype related to PTEN loss. In Pten null prostate epithelium, the gene restoration to wild type resulted in reduced cell proliferation and a rise in cell apoptosis (15). The assessment of molecular changes caused by homozygous PTEN deletion clearly identified important human related PCa genes such as NKX3.1 gene (17). These findings emphasize the cooperative effects of PTEN as a tumor suppressor gene and prostate-specific expressed NKX3.1 in PCa development (15–17).

## CONCLUSIONS

In the present study, we evaluated the expression of NKX3.1 and PTEN genes in clinical prostate samples to verify the correlation between the expression level of these two genes in human BPH and PCa. Our data confirmed the results from the previous studies regarding a concurrent decrease in both NKX3.1 and PTEN expression in prostate tumors. Moreover, statistical data analysis indicated a significant correlation between the expression level of the two genes in both BPH and PCa samples. This study further confirmed that these two genes may be considered as potential biomarkers in PCa besides other prostate biomarkers (2, 4).

However, we have not studied the expression of the proteins encoded by these genes. Therefore, it is highly favorable for future studies to evaluate the expression of both genes at protein level. To our knowledge, this is the first study to assess the simultaneous changes in the expression of PTEN and NKX3.1 in clinical PCa samples. According to our results, PTEN loss not only contributes to the reduced expression of NKX3.1 but also to prostate tumorogenesis. On the other hand, the link between the expression levels of PTEN and NKX3.1 genes could be implemented for the design of novel therapeutics for human PCa.
